# A customized Web portal for the genome of the ctenophore *Mnemiopsis leidyi*

**DOI:** 10.1186/1471-2164-15-316

**Published:** 2014-04-28

**Authors:** R Travis Moreland, Anh-Dao Nguyen, Joseph F Ryan, Christine E Schnitzler, Bernard J Koch, Katherine Siewert, Tyra G Wolfsberg, Andreas D Baxevanis

**Affiliations:** 1 Genome Technology Branch, Division of Intramural Research, National Human Genome Research Institute, National Institutes of Health, 50 South Drive, Bethesda, MD 20892, USA; 2 Whitney Laboratory for Marine Bioscience, University of Florida, St. Augustine, FL 32080, USA

**Keywords:** *Mnemiopsis leidyi*, Genome browser, Customized Web portal, Gene wiki

## Abstract

**Background:**

*Mnemiopsis leidyi* is a ctenophore native to the coastal waters of the western Atlantic Ocean. A number of studies on *Mnemiopsis* have led to a better understanding of many key biological processes, and these studies have contributed to the emergence of *Mnemiopsis* as an important model for evolutionary and developmental studies. Recently, we sequenced, assembled, annotated, and performed a preliminary analysis on the 150-megabase genome of the ctenophore, *Mnemiopsis*. This sequencing effort has produced the first set of whole-genome sequencing data on any ctenophore species and is amongst the first wave of projects to sequence an animal genome *de novo* solely using next-generation sequencing technologies.

**Description:**

The *Mnemiopsis* Genome Project Portal (http://research.nhgri.nih.gov/mnemiopsis/) is intended both as a resource for obtaining genomic information on *Mnemiopsis* through an intuitive and easy-to-use interface and as a model for developing customized Web portals that enable access to genomic data. The scope of data available through this Portal goes well beyond the sequence data available through GenBank, providing key biological information not available elsewhere, such as pathway and protein domain analyses; it also features a customized genome browser for data visualization.

**Conclusions:**

We expect that the availability of these data will allow investigators to advance their own research projects aimed at understanding phylogenetic diversity and the evolution of proteins that play a fundamental role in metazoan development. The overall approach taken in the development of this Web site can serve as a viable model for disseminating data from whole-genome sequencing projects, framed in a way that best-serves the specific needs of the scientific community.

## Background

Ctenophores are an important group of early-branching metazoans that are essential for understanding the evolution of multicellular animals, the relationship between genomic complexity and morphological complexity, and the molecular basis for the evolution of novel cell types such as epithelia, neurons, muscle, and stem cells. One ctenophore species that has received particular attention is *Mnemiopsis leidyi*, which is native to the coastal waters of the Atlantic Ocean. Studies in *Mnemiopsis* have advanced our understanding of a number of important biological processes such as regeneration, axial patterning, and bioluminescence [1-3]. As such, *Mnemiopsis* has emerged as an important model organism for understanding the immense diversity and complexity seen in the early evolution of animals.

Despite the importance of *Mnemiopsis* as an emerging model organism, there were no high-quality genome-scale sequence data available for any ctenophore species until recently. To address this dearth of genome-scale sequence data, we recently completed the sequencing, assembly, annotation, and preliminary analysis of the 150-megabase genome of *Mnemiopsis leidyi*[4]; these data will serve as an invaluable resource for the growing community of developmental, evolutionary, and marine biologists studying important questions regarding early branching metazoan biology. Initial studies utilizing these sequence data have contributed to our understanding of the evolution of gene families [5-7], signaling pathways [8,9], protein domains [10], miRNAs [11], and genes involved in the production and detection of light [12]. The availability of these data also has provided a solid foundation for studies aimed at resolving the question of the phylogenetic position of the ctenophores [4].

In recent years, databases have been created to house whole-genome sequencing data from several emerging model organisms. Genomic data and annotation are typically made accessible via public genome portals at sequencing centers such as the US Department of Energy’s Joint Genome Institute (JGI) [13] and the Broad Institute [14], while other groups have developed Web-based genomic database resources that provide additional analysis tools [15] and browsing options [16] to increase the utility of the data. Still others have implemented genomic resources that offer the scientific community access to genomic annotation and actively seek user contributions [17]. Ideally, for each organism with a sequenced genome, there would be a single centralized resource where most (if not all) data retrieval and analysis could take place; this kind of resource would include, at a minimum, the ability to search, browse, and download sequence and annotation data, visualize genomic data via a Web-based browser tool, and encourage the active engagement of the scientific community in maintaining a wiki-style resource for capturing supplementary annotations of gene models and predicted proteins. Moreover, this kind of resource would (and should) be developed and maintained by researchers in the model organism community who intimately understand the needs of their scientific colleagues, presenting the data in an intuitive, user-friendly and concise manner despite its sheer volume and complexity.

In our own experience advising groups who have undertaken whole-genome sequencing projects, we have found that many of these groups do not have ready access to the kind of programming resources needed to implement some of the more “advanced” database solutions currently available. With that in mind, and to facilitate the creation of the kind of centralized genomic data resource described above, we set out to develop a generalized framework that strikes a reasonable balance between ease of implementation and documented structure, without the additional constraints posed by some publicly available database schemas.

Here, we describe the development and features of a comprehensive Web-based data portal for navigating the recently completed genome sequence of *Mnemiopsis leidyi* (http://research.nhgri.nih.gov/mnemiopsis/). The *Mnemiopsis* Genome Project Portal (or “MGP Portal”) is a biologist-centric resource designed with a particular emphasis on usability, intuitive navigation, and clarity. Some key features of the MGP Portal include the ability to retrieve selected nucleotide and protein sequences, the availability of whole-genome datasets for download, an integrated BLAST utility for sequence comparisons, a genome browser tool, a gene-centric wiki, and “phylogenetically informed” gene ortholog clusters mapped to human KEGG pathways. Furthermore, the scope of data accessible through this Web site goes well beyond the sequence data available at GenBank, providing other key biological information such as Gene Ontology term assignments and data from pathway and protein domain analyses. In addition, we offer a set of Perl modules that can be utilized by other scientists as a generalized framework for implementing a gene page in MediaWiki, as well as a customizable genome browser for visualizing large-scale genomic data using JBrowse.

## Construction and content

### Displaying customized genome annotation

**Figure 1:**
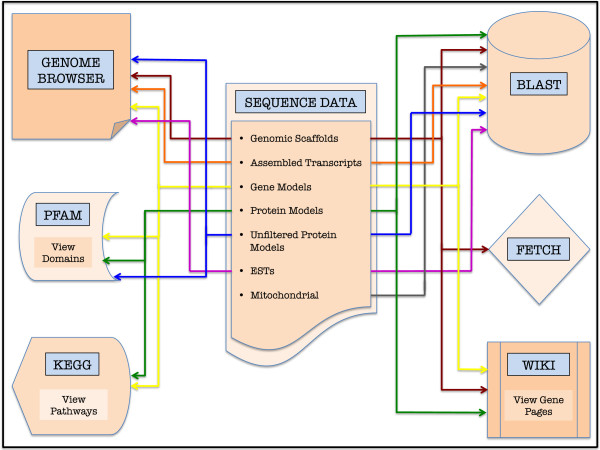
**A visual representation of the *Mnemiopsis *Genome Project (MGP) Portal depicting the flow of *Mnemiopsis *sequence data into accessible internal data visualization and annotation tools.** Colored arrows correspond to individual data types listed in the Sequence Data box (center). For example, the flow of ‘Assembled Transcripts’ data (*e.g.*, Cufflinks- and Trinity-assembled RNA-seq transcripts) is represented by the orange arrows; these data can be viewed both as tracks in the Genome Browser and/or utilized as a BLAST database.

A major goal during the development of the MGP Portal was to create a reproducible workflow for the creation of well-annotated and visually accessible genome repositories (Figure 1). To that end, we have adopted the JavaScript-based JBrowse [18] as the engine for our *Mnemiopsis* Genome Browser, resulting in a clean and responsive user interface for viewing the genome assembly, gene models, and all supporting data. We have developed a number of tools in the Perl scripting language to convert sequence and annotation data into a format accepted by JBrowse (version 1.11.1), which are included as Additional files 1, 2 and 3. In addition, we provide a Perl script that facilitates the creation of MediaWiki pages that display nucleotide and protein sequences, exonic genomic coordinates, and PFAM domains (Additional file 4). The actual creation of genome assemblies and annotation files is outside the scope of this manuscript but is outlined in detail elsewhere [4].

To build feature tracks, JBrowse requires properly formatted input files. While newer versions of JBrowse released subsequent to the creation of the *Mnemiopsis* Genome Browser are able to accept a slightly expanded number of inputs (including specific relational database dumps), the generic feature format version 3 (GFF3) flat file was the input type we adopted, a format that continues to be supported by JBrowse at the time of this writing (version 1.11.1). Adoption of the GFF3 format greatly facilitated the processing of multiple output file types produced by a number of data analysis programs. To begin, the *scaffoldToGFF3.pl* script (Additional file 1) can be used to reformat scaffold sequences from FASTA to GFF3. Two parameters (–i and –o) are required, specifying the input scaffold file and desired output directory, respectively. Two optional parameters (–l and –n) are also available. The first should be called if the gap-indication letter in the input is different from ‘N’ , accepting a single character (*e.g.*, X) as a substitute, while the second parameter can be called in order to specify which feature-generating program was used. The script creates a GFF3 file for each sequence in the input scaffold FASTA file and is able to handle both gapped [*e.g.*, the scaffold (SCF) track] and repeat-masked [*e.g.*, the repeat-masked (MASK) track] regions in a scaffold. Next, *evmToGFF3.pl* (Additional file 2) parses a GFF3-formatted output file created by EvidenceModeler (EVM) [19] by collecting data about the start and end positions of predicted genes, using this information to create well-formed GFF3 files. The script accepts several additional parameters; the input EVM file (–i) and desired output directory (–o) must be set, while the third and optional parameter (–n) is, again, the name of the feature-generating program (*e.g.,* EVM). The third module is called *cufflinksToGFF3.pl* (Additional file 3) and is used to parse predicted transcript assemblies from the GTF-formatted file created by Cufflinks [20]. The *cufflinksToGFF3.pl* script has the same behavior for transcript location as *evmToGFF3.pl* has for predicted gene location, and accepts the same three parameters (–i, –o, and –n).

To import GFF3 data into JBrowse for display as custom tracks in the main genome window, a series of three JBrowse-supplied Perl scripts (*prepare-refseqs.pl, biodb-to-json.pl,* and *generate-names.pl*) need to be executed using appropriate parameters and a system-specific configuration file, the details of which can be found in the tutorials on the JBrowse Web site (http://www.jbrowse.org). A number of custom CGI scripts were written to create the hyperlinks connecting features in JBrowse to the various sources of gene data.

A Perl script named *create_wiki_page.pl* has been used to create MediaWiki pages for displaying genomic data (Additional file 4). Here, we provide a sample wiki page that takes FASTA-formatted nucleotide and protein sequences, GFF3 files containing exonic coordinates, and an hmmscan output file containing information on Pfam-A domains as input. The *create_wiki_page.pl* script requires five parameters. The first three parameters specify a set of input files, including a nucleotide FASTA file (-n), a protein FASTA file (-a), and a Pfam-A file (-p). The remaining parameters specify a directory containing the input GFF3 files containing the exonic coordinates (-d) and an output directory (-o). In addition, we show a PHP command line that imports a wiki page into MediaWiki:php/$WIKIHOME/maintenance/importTextFile.php - -user=USER wikipage.out The wikipage.out file is created by the *create_wiki_page.pl*.

Researchers interested in creating a customized genome browser or gene wiki for visualizing genome-related data are encouraged to utilize the aforementioned scripts (Additional files 1, 2, 3 and 4), as their implementation satisfies the fundamental requirements of both JBrowse and MediaWiki. Furthermore, these modules may serve as a useful framework for both the development of gene wikis and more advanced genome browser tracks as new JBrowse applications are created to visualize genomic data.

### User interface and genome browser implementation

*Mnemiopsis* sequences are stored as individual text files, and several Perl scripts were written to retrieve these as single combined files. Multiple sequences can be downloaded via an HTML interface that was developed using a series of CGI/Perl scripts. The *Mnemiopsis* BLAST tool was implemented using ViroBLAST [21] and runs on an Apache Web server (Additional file 5: Figure S1). The ViroBLAST tool was written in PHP, a server-side scripting language [22], and Perl [23], applying the stand-alone blastall program downloaded from NCBI [24]. The *Mnemiopsis* BLAST databases were created using formatdb. PHP was used to parse the BLAST output created by ViroBLAST to generate the formatted BLAST results, including the internal links to the Genome Browser, the Gene Wiki pages and the Fetch Scaffold Tool. MediaWiki (version 1.19.11), written in PHP and Perl, was used for the Gene Wiki implementation. Perl was also used to create data for displaying on the Gene Wiki pages. The KEGG pathways pages were developed using JavaScript to display KEGG identifier, pathway, and gene symbol search lists. CGI/Perl scripts were used for KEGG pathway search functions and data downloading utilities (Additional file 6: Figure S2). Python (version 2.6) [25] scripts were used to search the Pfam domains, identified using hmmscan [26] from the HMMER suite, and CGI and JavaScript were used to display the Pfam domain search results (Additional file 7: Figure S3).

## Utility and discussion

### *Mnemiopsis* BLAST tool

**Figure 2:**
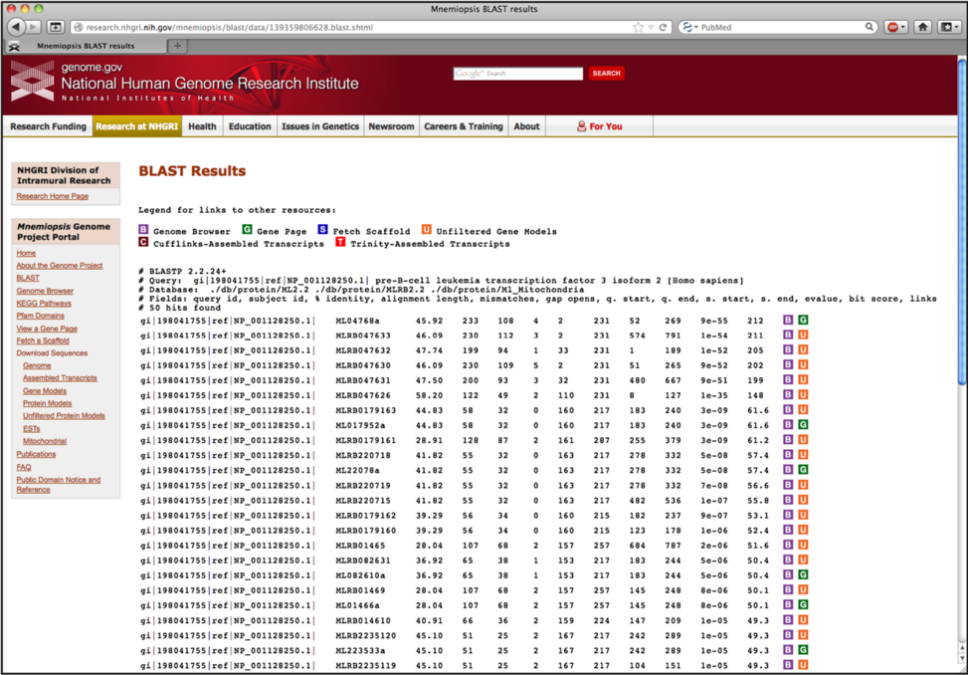
**The *Mnemiopsis *BLAST implementation provides users an intuitive Web interface for performing sequence similarity searches.** Shown are tab-delimited BLASTP results from a single human PAX3 protein, providing links to relevant sequence entries in the *Mnemiopsis* Genome Browser (purple ‘B’ box), individual Gene Wiki pages (green ‘G’ box), and Unfiltered Protein Models (orange ‘U’ box).

One feature of the *Mnemiopsis* Genome Project Portal is a customized stand-alone Web-based BLAST interface for performing nucleotide and amino acid sequence similarity searches (Additional file 5: Figure S1). ViroBLAST was used to implement our *Mnemiopsis* BLAST tool, producing an organized, manageable output that is easy to parse and navigate. Users may input their FASTA-formatted query sequences directly into the search box or upload sequence files from their computer. The customary set of BLAST programs is available, including BLASTN, BLASTP, BLASTX, TBLASTN, and TBLASTX. Nucleotide sequence databases include the *Mnemiopsis* genomic scaffolds (Main Scaffolds), consensus gene prediction models (Gene Models 2.2) and Unfiltered Gene Models (unincorporated predictions) described in Ryan *et al.*[4], all publicly available *Mnemiopsis* ESTs and mRNAs from GenBank (Public ESTs), the *Mnemiopsis* mitochondrial genome [27], Cufflinks-assembled RNA-seq transcripts, and Trinity-assembled RNA-seq transcripts [28]. The protein sequence databases available through the MGP Portal include the translated proteins derived from the *Mnemiopsis* consensus gene prediction models (Protein Models 2.2), the unincorporated *Mnemiopsis* proteins derived from unincorporated gene prediction models (Unfiltered Protein Models), and the computationally derived *Mnemiopsis* mitochondrial proteins. Additionally, a user may create a customized user-defined BLAST database by uploading a file containing FASTA-formatted nucleotide or protein sequences of interest. BLAST output results feature customized color-coded boxes linked directly to relevant internal annotation resources, including the *Mnemiopsis* Genome Browser [B], the wiki-based *Mnemiopsis* Gene Pages [G], the Scaffold Fetch Tool [S], Unfiltered Gene Models [U], Cufflinks-assembled transcripts [C], and Trinity-assembled transcripts [T] (Figure 2).

### Browsing the *Mnemiopsis* genome

**Figure 3:**
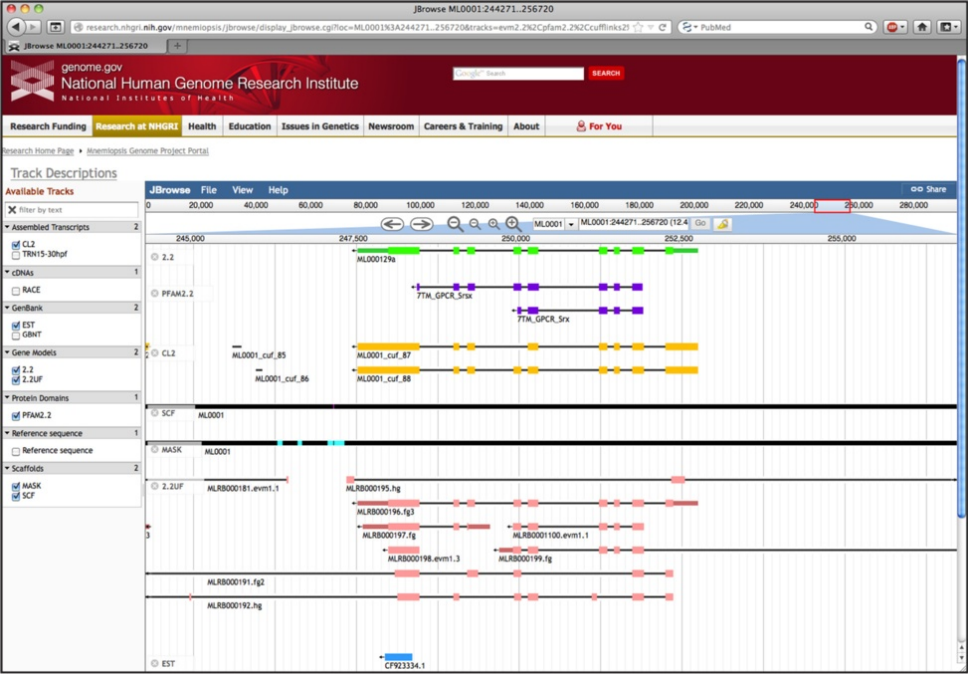
**Several customized tracks can be displayed on the *Mnemiopsis *genome browser, implemented in JBrowse.** Here, we present a predicted protein model (ML000129a) containing G-protein-coupled receptor domains, as evidenced by the PFAM2.2 track. Transcripts assembled from RNA-seq reads support the predicted protein model as depicted in the CL2 track. Transcripts are presented as gray arrows indicating the orientation of the transcript. Exons are presented as light-colored solid bars and untranslated regions (both 5’ and 3’) are darker-shaded bars. Assembled genomic scaffolds (SCF) are depicted as solid black tracks with intermittent gaps shaded bright pink. The MASK track also appears as a solid black bar highlighted with blue in the genomic regions that have been repeat-masked using VMatch. Other tracks shown include an EST and several unfiltered protein prediction models (2.2UF). Additional tracks are described in the main text.

One of our primary objectives in developing the MGP Portal is to provide a graphical tool for the scientific community to visualize the various types of *Mnemiopsis* genome data currently available. Using the built-in JBrowse genome browser, users can view a variety of data tracks, such as the *Mnemiopsis* genome assembly, gene prediction models, and RNA-seq data (Figure 3). Several customized JBrowse tracks are available for viewing, including consensus gene prediction models (labeled 2.2 in the browser), *Mnemiopsis* RNA-seq reads assembled into transcripts using Cufflinks (CL2) and Trinity (TRN15-30 hpf), publicly available *Mnemiopsis* ESTs from GenBank (EST), publicly available *Mnemiopsis* mRNAs from GenBank (GBNT), assembled genomic scaffolds (SCF), genomic regions that have been repeat-masked using V-Match (MASK) [29], experimentally verified *Mnemiopsis* RACE PCR transcripts (RACE), unincorporated gene prediction models (2.2UF), and non-redundant protein domains derived from Pfam hmmscan runs using the 2.2 and 2.2UF datasets and the six-frame translations of the *Mnemiopsis* genome (PFAM2.2).

The JBrowse display is organized by genomic scaffold. Scaffolds are named using a six-character convention (*e.g.*, ML*nnnn*); the ML designates the species (*Mnemiopsis leidyi*), and the individual scaffolds are numbered from 0001 to 5100 (*e.g.*, ML0001). Gene identifiers (*e.g.*, ML000129a) start with a prefix indicating the scaffold on which the gene is located (in this example, ‘ML0001’), followed by a non-padded integer that is unique in combination with the scaffold identifier (in this case, ‘29’) and ending with a lower-case letter that specifies the gene isoform (in this case, ‘a’). Data displayed in the browser can be searched using a variety of *Mnemiopsis* identifiers. A scaffold-based query takes the user directly to that scaffold, while a gene-based search goes to that gene’s location on the appropriate scaffold. A user may also search the genome browser using a *Mnemiopsis* GenBank mRNA identifier, an EST identifier, or a Pfam-A domain name (*e.g.,* AF293700.1, FC475136, or “Glyco hydro 20”, respectively). The PFAM2.2 track was created by running hmmscan against the Protein Models 2.2, the Unfiltered Protein Models, and the six-frame translations of the *Mnemiopsis* genome. Scaffold coordinates are displayed across the top of the browser window. Navigation options can be found beneath the coordinate bar, including the zoom tool and left-right arrows. Users can also refine the displayed region by entering the scaffold coordinates into the search box to the right of the navigation options.

Genome browser tracks are described in the Track Descriptions link above the left sidebar. The consensus *Mnemiopsis* gene prediction models (track 2.2) are presented by default. Additional track can be added by clicking on the appropriate track box on the left sidebar of the main view window. All track options are displayed in a given scaffold window even when there are no annotated features in that particular region. Features are represented as black arrows, with the direction of the arrow indicating the orientation. Exons are presented as light-colored solid bars (*e.g.*, green for 2.2 and pink for 2.2UF), while untranslated regions (both 5’ and 3’) are rendered using darker-shaded colors. Assembled genomic scaffolds (SCF) are depicted as solid black tracks with intermittent bright pink gaps. The MASK track appears as a solid black bar, with blue highlighting the regions that were repeat-masked using VMatch (Figure 3). The Reference sequence track depicts the scaffold sequence, but only when the display is fully zoomed in.

### *Mnemiopsis* genes in KEGG pathways

**Figure 4:**
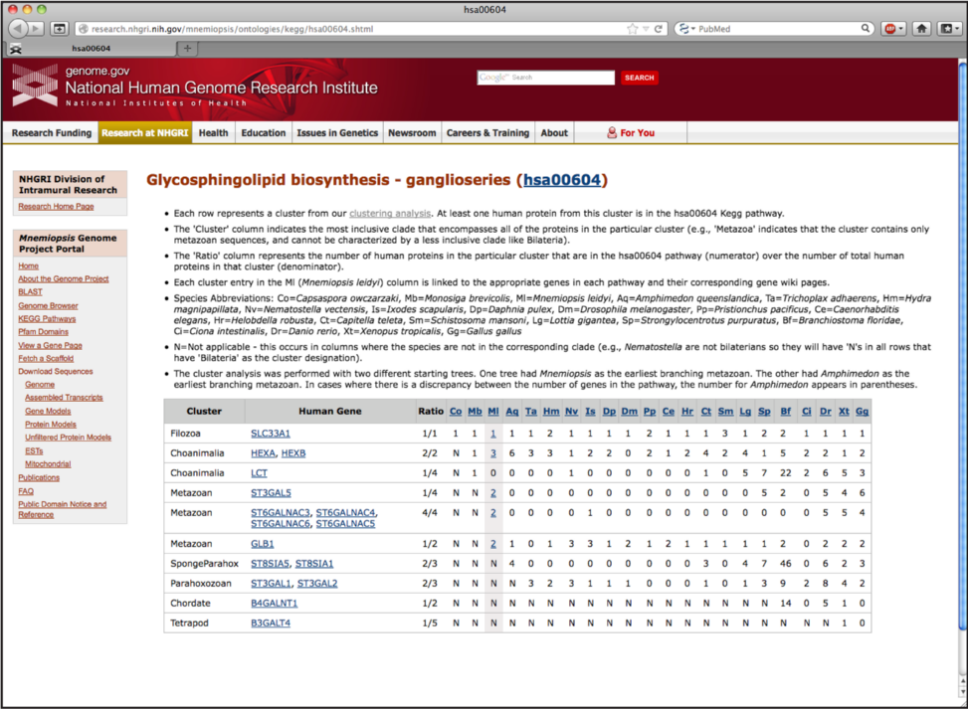
**Human KEGG pathways containing genes with a *Mnemiopsis *homolog are presented as pathway-specific ortholog cluster matrices.** Each row in the glycosphingolipid biosynthesis pathway represents a cluster from our clustering analysis. The ‘Cluster’ column indicates the most inclusive clade that encompasses all of the proteins in the cluster. The ‘Ratio’ column represents the number of human proteins in a given cluster that are found in the pathway over the total number of human proteins in that cluster. *Mnemiopsis* (Ml) entries are shaded in gray and hyperlinked to their respective Gene Wiki pages.

Previously, we identified gene clusters that contain likely orthologs of both *Mnemiopsis* and human proteins [4]. In order to gain some insight into the function of individual *Mnemiopsis* genes, we used this information to assign individual *Mnemiopsis* genes to human KEGG pathways. We converted Ensembl identifiers for all human genes from our phylogenetically informed clusters of orthologous genes [4] to Entrez Gene IDs using a file from the Entrez Gene FTP site [30]; we then used EASE [31] to link Entrez Gene IDs to KEGG pathways. These data can be accessed by following the KEGG Pathways link found in the left sidebar of most MGP Portal pages. Human KEGG pathways containing genes with a *Mnemiopsis* ortholog are searchable by selecting a KEGG identifier (*e.g.,* hsa00604), a pathway name (*e.g.,* glycosphingolipid biosynthesis), or a gene symbol (*e.g.,* SLC33A1) from their respective lists, or by using the KEGG pathways search box (Additional file 6: Figure S2). The results are presented as pathway-specific ortholog cluster matrices (Figure 4).

For each KEGG pathway, each row in the ortholog cluster table represents a cluster of orthologous genes from our clustering analysis [4]. For a cluster to be included in the table, at least one human gene from that cluster must be present in the KEGG pathway represented. In most cases, a row consists of one or more human genes that belong to the selected KEGG pathway, along with the computationally predicted orthologs from *Mnemiopsis* and 21 other model organisms. The ‘Cluster’ column indicates the most inclusive phylogenetic clade that encompasses all of the genes in the particular cluster (*e.g.,* ‘Metazoa’ could indicate that the cluster contains genes from both bilaterians and cnidarians and thus cannot be characterized by a less inclusive clade, such as ‘Bilateria’). Each human gene is hyperlinked to its Entrez Gene entry. The ‘Ratio’ column represents the number of human genes in the particular cluster that are in a given pathway (numerator) over the number of total human genes in that cluster (denominator). The higher the ratio, the more likely the non-human orthologs in the cluster are involved in the pathway. The numbers in the columns under each species abbreviation indicate the number of genes from that species that are in that cluster. Each number in the ‘Ml’ (*Mnemiopsis leidyi*) column is linked to the appropriate *Mnemiopsis* gene ID(s) and corresponding Gene Wiki pages.

### Pfam domains in *Mnemiopsis* proteins

Another way to characterize the *Mnemiopsis* genes is to determine the protein domains that they encode. We used hmmscan from the HMMER suite (HMMER 3.0; March 2010) to search the Protein Models 2.2 and Unfiltered Protein Models for domains from the Pfam-A database (version 25). The gathering threshold (cut_ga) option was used to ensure conservative domain prediction. The Pfam Domains link on the home page of the MGP Portal takes the user to a query page, where researchers can specify a given Pfam-A domain by name or accession number, then search for *Mnemiopsis* genes that contain that domain (Additional file 7: Figure S3). The results are displayed as a list of protein models, listed by gene identifier, and the number of query domains found in those protein models. Additionally, a user may download FASTA-formatted Pfam-A domain sequences from the resulting list by clicking on the check boxes next to the sequence(s) of interest, selecting either Pfam-A domain only or the full-length domain-containing protein from the pull-down menu, and clicking ‘Get’.

### *Mnemiopsis* Gene Wiki

**Figure 5:**
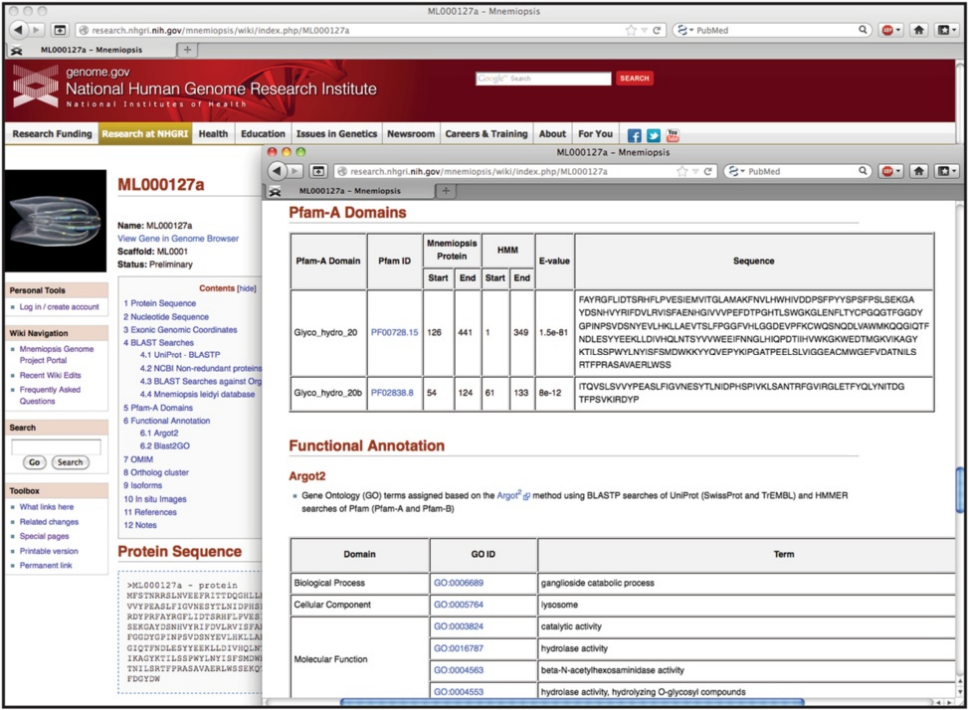
**Each record in the Gene Wiki represents a single *Mnemiopsis *gene (ML000127a) and provides the following annotation: nucleotide and protein sequences, coding exonic genomic coordinates, and pre-computed BLAST hits from numerous organisms displaying the top hits for each protein.** The inset illustrates additional annotations available through the Gene Wiki pages, including those regarding Pfam-A domains and GO terminology (functional annotation).

In an effort to engage the collective expertise of the scientific community, we have implemented a collaborative wiki (MediaWiki version 1.19.11) for the *Mnemiopsis* gene complement. The *Mnemiopsis* Gene Wiki is accessible from the left sidebar of most pages and is searchable either by selecting a *Mnemiopsis* gene identifier (*e.g.,* ML00011a) from the drop-down menu or by manually entering an identifier in the appropriate search box. Users can also access these pages by clicking on a gene in the 2.2 track of the genome browser. Each record in the Gene Wiki represents a single *Mnemiopsis* gene and provides the following annotation: nucleotide and protein sequences, coding exonic genomic coordinates, pre-computed BLAST hits from numerous organisms displaying the top hits for each protein, the top non-self BLAST hit to *Mnemiopsis*, Pfam-A domains, Gene Ontology (GO) functional annotations, human disease genes from Online Mendelian Inheritance in Man (OMIM), and a table of ortholog clusters formed by phylogenetically informed clustering methods [4] (Figure 5). In addition, controlled editable sections have been included that permit (and encourage) the scientific community to provide further gene annotation for isoforms, *in situ* images, references, and other notes for each gene. Users interested in supplementing our gene model annotation at the *Mnemiopsis* Gene Wiki pages must first create an account and log in prior to submitting their contributions. In-house subject matter expert data curators are notified by e-mail following the creation of a new user account or an edit to an existing Gene Wiki record. Any content changes or additions to the Gene Wiki are thoroughly evaluated by these data curators and are made public subject to their approval.

Pre-compiled BLAST hits are enumerated in tabular form. Each *Mnemiopsis* protein was compared to the UniProt and NCBI non-redundant protein databases (nr) using BLASTP. The results display the hit number, the accession numbers, *E*-values, and brief descriptions of the top four hits (lowest *E*-values). Accession numbers are linked to relevant corresponding entries at UniProt and GenBank. The *E*-values are hyperlinked to the pairwise BLAST alignments.

Each *Mnemiopsis* protein was also compared to sequence data from developmentally relevant organisms, including *Homo sapiens*, *Drosophila melanogaster*, *Capitella teleta*, *Amphimedon queenslandica*, *Nematostella vectensis*, *Hydra magnipapillata*, *Trichoplax adhaerens*, *Monosiga brevicollis*, *Salpingoeca rosetta*, *Capsaspora owczarzaki*, fungi, plants, and non-eukaryotes. The top hits for BLASTP and TBLASTN results, falling below an established *E*-value threshold (*E*-value ≤ 1 × 10^-6^), are displayed along with their gene or protein identifiers, *E*-values, and description of the best hits. For the *Mnemiopsis* organismal database search, the gene identifier of the top non-self hit is displayed (and linked to its corresponding Gene Wiki page) along with the *E*-value for that alignment.

The Gene Wiki also contains a section displaying the Pfam-A domains that are encoded by the protein. The Pfam identifier, domain architectures, sequence start and end coordinates, HMM start and end coordinates, *E*-values, and domain sequence are displayed for each Pfam-A domain. All Pfam identifiers are hyperlinked to their corresponding entries on the Pfam Web site [32]. To further assist in classification, GO terms are presented for each gene, with GO terms assigned using the Argot2 [33] method. Functional annotations derived using Blast2GO [34] are also presented for each gene.

### Retrieving single scaffold sequences

**Figure 6:**
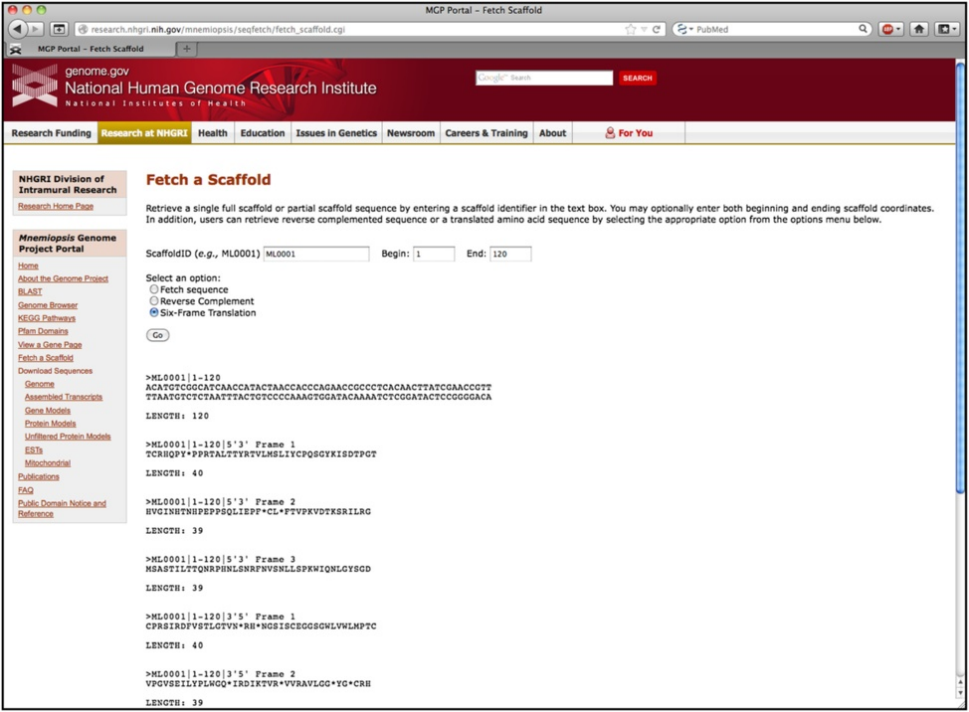
**The *Mnemiopsis *Fetch Tool is used to retrieve a single or partial scaffold, its reverse complement or the six-frame protein translation.** Displayed above is the output of a queried partial genomic scaffold for ML0001 showing the specified genomic region of interest and its six-frame protein translations (the first five translations are depicted).

The genomic sequence of a single (or partial) scaffold can be retrieved using the Fetch Scaffold Tool. A user can download a FASTA-formatted full genomic scaffold sequence by following the Fetch a Scaffold link in the left sidebar of the MGP Portal home page, entering a scaffold identifier (*e.g.,* ML0001) in the search box, and selecting the Fetch sequence option (Figure 6). Partial scaffold sequences can be retrieved by adding scaffold coordinates to the above query. Alternatively, users can retrieve the reverse complement or six-frame protein translation of the scaffold by selecting the appropriate option.

### Downloading full or partial datasets

As noted above, one of our primary objectives in developing the MGP Portal is to simplify the dissemination of all *Mnemiopsis* sequence data to the scientific community at large. To that end, we have provided users a direct method for obtaining entire *Mnemiopsis* datasets as compressed text files. The following complete sequence datasets can be downloaded by clicking on the appropriate links located in the left sidebar: the *Mnemiopsis leidyi* genome assembly (5,100 scaffolds), the full set of *Mnemiopsis* Gene Models (16,548 genes), the *Mnemiopsis* Protein Models (16,548 proteins), the *Mnemiopsis* Unfiltered Protein Models (60,006 proteins), all publicly available *Mnemiopsis* EST sequences from NCBI (15,752 ESTs), and the full *Mnemiopsis* mitochondrial genome and protein sequences [27] (11 proteins; Table 1). Optionally, users may enter a known *Mnemiopsis* sequence identifier (*e.g.,* ML0001 or ML00011a) in the search box or select from a list of identifiers to retrieve a single scaffold, gene model, protein model, or EST of interest.

**Table 1:** ***Mnemiopsis leidyi *** **complete sequence datasets available for download from the *Mnemiopsis *** **Genome Project Portal**

**Dataset**	**Number of sequences**
Genome assembly (scaffolds)	5,100
Gene models	16,548
Protein models	16,548
Unfiltered protein models	60,006
ESTs	15,752
Mitochondrial genome	1
Mitochondrial proteins	11

### Demonstrating the MGP Portal’s utility: a worked example

The MGP Portal was developed to facilitate research that would benefit from the availability of genomic information from this emerging model organism and, to this end, it includes a number of intuitive data analysis tools. To illustrate this point, consider the case of a developmental biologist studying the human TALE class homeobox gene family (*e.g.,* PBX3; [GenBank:NP_001128250.1]) who may be interested in comparing these sequences against (or predicting novel) *Mnemiopsis* homeodomain orthologs. A straightforward approach to addressing this question would be to run a BLASTP search of the PBX3 protein sequence against the *Mnemiopsis* Protein Models (2.2) database. The *Mnemiopsis* BLAST results display a number of high-scoring candidate proteins that can be further evaluated for properties characteristic of TALE class homeodomains (*e.g.,* a TALE-type homeobox; Figure 1).

**Figure 7:**
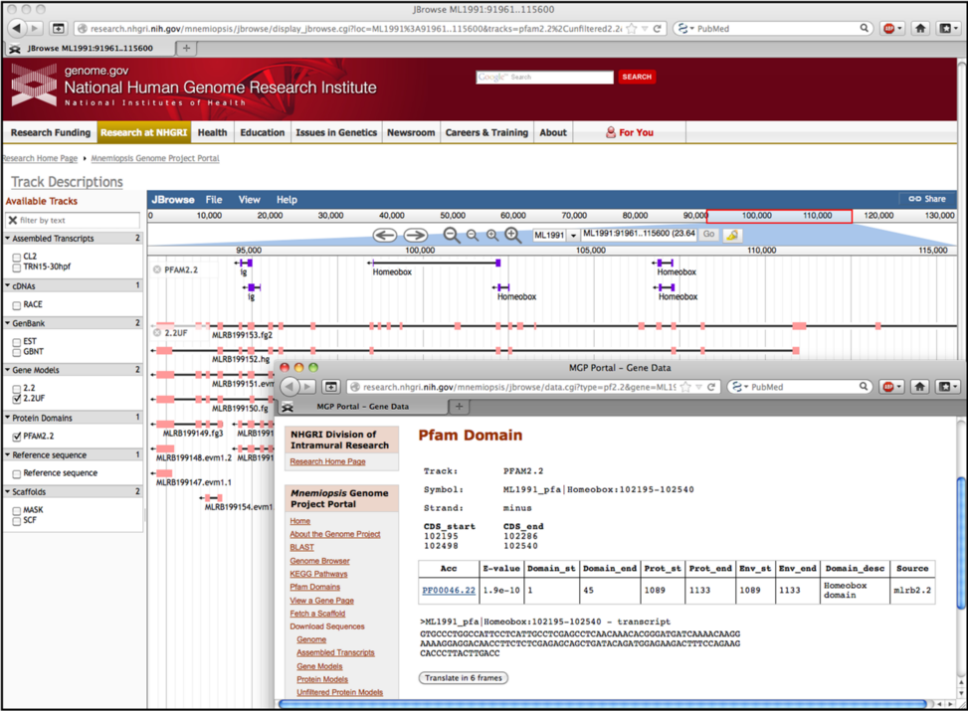
**Selecting the Genome Browser link (purple ‘B’ box from Figure ** 1**) from a BLASTP result entry of queried known homeodomains against the complete *Mnemiopsis *Unfiltered Protein Models directs the user to the Genome Browser displaying the applicable *Mnemiopsis *transcript model.** Shown above the 2.2UF track in the browser is the PFAM2.2 track displaying evidence of a homeobox in the targeted region. Clicking on the ‘Homeobox’ link in the PFAM2.2 track opens a new browser window displaying the Pfam-A domain (ML1991_pfa) prediction results derived from a pre-compiled hmmscan run using HMMER. This Pfam-A domain record provides the genomic location of the Pfam-A domain, the genomic coordinates, transcript and protein sequences, and the hmmscan output for the homeodomain prediction.

Alternatively, another biologist may be interested in searching for novel *Mnemiopsis* homeodomains, using sequence data from a closely related organism such as *Amphimedon* as the basis for their search. One approach would be to retrieve a complete set of known *Amphimedon* homeodomain proteins through an NCBI Entrez query (Search < Protein > for: “homeodomain AND Amphimedon[ORGN]”, which yields 31 known *Amphimedon* homeodomain proteins at the time of this writing). FASTA sequences can then be copied and pasted into the *Mnemiopsis* BLAST search window or uploaded as a file from a local computer. A unique feature of the MGP Portal BLAST implementation includes access to the Unfiltered Protein Models database, which contains the complete unincorporated (unfiltered) protein dataset derived from the *Mnemiopsis* gene prediction and annotation process. A BLASTP search against the Unfiltered Protein Models database provides additional information about possible alternate transcripts and isoforms that were screened and filtered out during the initial annotation process. These unfiltered protein models can be placed into genomic context by following the color-coded *Mnemiopsis* Genome Browser [B] links in the far right-hand column of the BLAST results. The browser provides direct access to additional annotation, including a graphical representation of the Pfam-A domain that overlaps the protein model, as well as links to the sequence of the Pfam-A domain (Figure 7).

Similarly, a researcher may also want to use their own custom scripts or external computational tools to further-explore the available *Mnemiopsis* data sets. In such a case, the Download Sequence links in the MGP Portal can be used to download both the Protein Models and the Unfiltered Protein Models for analysis with tools from the HMMER suite [26] (*e.g.,* hmmsearch Homeobox.hmm ML2.2aa > ML_novel_HDs). Predicted domains with *E*-values below an inclusion threshold (*e.g., E*-value < 0.05) could then be considered candidate homeodomains for further evaluation.

## Conclusions

The *Mnemiopsis* Genome Project Portal is intended as a resource for investigators from the scientific community to obtain genomic information on *Mnemiopsis* through an intuitive and easy-to-use interface; it also serves as a model for researchers undertaking the development of such a customized genome portal themselves. There are a number of comprehensive data portals available for well-established model organisms (*e.g.*, FlyBase). However, as we searched for model Web sites from which to draw inspiration for the MGP Portal, we found that many repositories for next-generation sequencing data are simply Web sites with lists of links to raw sequence data accompanied by minimal annotation, or were non-intuitive and difficult to navigate. Based on this experience, we felt that the selection and utilization of essential resources to systematically manage and disseminate the considerable amounts of data generated by these sequencing projects was imperative. Thus, the presentation and conveyance of such a genome Web portal should be intuitive, user-friendly, and concise. It is within this framework that we present the MGP Portal as such a resource for the recently completed genome sequence of *Mnemiopsis leidyi*, and we are hopeful that this resource will inspire other groups as they create Web portals of their own.

It was our intent during the development of the MGP Portal to develop a resource to maximize usability while presenting a comprehensive series of datasets not available elsewhere. Recognizing the difficulties and lessons learned from the development of such a resource, and in our continued effort to further communicate our shared experiences to the scientific community at large, we encourage other investigators to consider the proposed genome portal model and, as such, have included a series of scripts (Additional files 1, 2, 3 and 4) to facilitate the conversion of output files produced by various programs. Specifically, this series of scripts can be used to format annotation data for visualization within a customized genome browser and a wiki.

As described above, the MGP Portal contains sequence-based information and several customized utilities not available elsewhere, increasing the utility of the data generated by our group in the course of our *Mnemiopsis* whole-genome sequencing project [4]. The genome browser tool provides an intuitive interface for users to visualize the various types of data available, including data resulting from our comprehensive annotation of the *Mnemiopsis* genome. Most importantly, many features of this site make it easy for users who do not have a background in bioinformatics to straightforwardly access information presented from a comparative genomics point-of-view, without having to perform many of the analyses themselves. For instance, our phylogenetically relevant gene clusters are mapped to human KEGG pathways, providing a clear phylogenetic perspective for any particular *Mnemiopsis* gene (or pathway of genes) of interest. In addition, users may contribute to our gene annotation efforts by adding isoforms, *in situ* images, or other notes to any Gene Wiki page using a secure login. We trust that the availability of these data will allow investigators from numerous fields (such as developmental, evolutionary, and marine biology) to advance their own research projects aimed at understanding phylogenetic diversity and the evolution of proteins that play a fundamental role in metazoan development.

## Availability and requirements

The *Mnemiopsis* Genome Project Portal is freely available at http://research.nhgri.nih.gov/mnemiopsis, with no barriers to access. Registration is only required if users wish to contribute data to the Isoforms, *In situ* Images, References, or Notes sections of any of the Gene Wiki pages.

## Competing interests

The authors declare that they have no competing interests.

## Authors’ contributions

JFR and ADB conceived the study. RTM, ADN, and TGW designed and developed the database with critical input from JFR, CES, and ADB. ADN wrote the code and implemented the interfaces and tools associated with the MGP Portal. RTM, JFR, CES, BJK, KS, and TGW performed the genome annotation and data analysis. RTM, JFR, CES, TGW, and ADB tested the Web application and tools and provided feedback. RTM and ADB wrote the manuscript, with input and suggestions from CES, JFR, and TGW. ADB directed the project. All authors read and approved the final manuscript.

## Supplementary Material

Additional file 1 **The *scaffoldToGFF3.pl *script reformats scaffold sequences from FASTA to GFF3.** The script creates a GFF3 file for each sequence in the input scaffold FASTA file and is able to handle both gapped [*e.g.*, the scaffold (SCF) track] and repeat-masked [*e.g.*, the repeat-masked (MASK) track] regions in a scaffold. Click here for file

Additional file 2 **The *evmToGFF3.pl *** **script parses a GFF3-formatted output file created by EvidenceModeler (EVM) by collecting data about the start and end positions of predicted genes, using this information to create well-formed GFF3 files.** Click here for file

Additional file 3 **The *cufflinksToGFF3.pl *** **script parses predicted transcript assemblies from the GTF-formatted file created by Cufflinks.** Click here for file

Additional file 4 **The *create_wiki_page.pl *script creates MediaWiki pages for displaying genomic data.** Here, we provide a sample wiki page that takes as input FASTA-formatted nucleotide and protein sequences, GFF3 files containing exonic coordinates, and an hmmscan output file containing information on Pfam-A domains. The output of this perl script is called wikipage.out. Click here for file

Additional file 5: Figure S1 The *Mnemiopsis* BLAST tool (implemented using ViroBLAST) schematic illustrates the available user-defined input and output formats, BLAST programs, and database options. BLAST databases are provided for both *Mnemiopsis* nucleotide (e.g., Mitochondrial genome) and protein [e.g., Protein Models (2.2)] data. Click here for file

Additional file 6: Figure S2 The KEGG Pathways search function permits users to search KEGG pathways containing human genes, using a *Mnemiopsis* homolog as the query. The relationships underling the search function are depicted as a series of associated flat files. A one-to-one relationship exists between the KEGG and PATHWAY tables and the PEP2SOURCE_ID and CLUSTERS tables. All other relationships are one–to-many or many-to-many. The CLUSTERING_ANALYSIS table is the final output representation of a KEGG Pathways query consisting of the combination of KEGG_ENTREZ_GENE, SPECIES, and CLUSTERS. Click here for file

Additional file 7: Figure S3 The PFAM Domains search function parses a series of flat files illustrated here as a relational framework. The PFAM Domains schema is represented as six attributes, with connectors indicating the nature of each applicable relationship. DOMAIN_ACCESSION and DOMAIN_NAME have a one-to-one relationship. All other relationships between PFAM attributes are many-to-many. Click here for file
