# Supplementary figures and images for: A customized Web portal for the genome of the ctenophore Mnemiopsis leidyi
Source: BMC Genomics. 2014 Apr 28;15:316. doi: 10.1186/1471-2164-15-316 (PMC4234515; doi:10.1186/1471-2164-15-316)

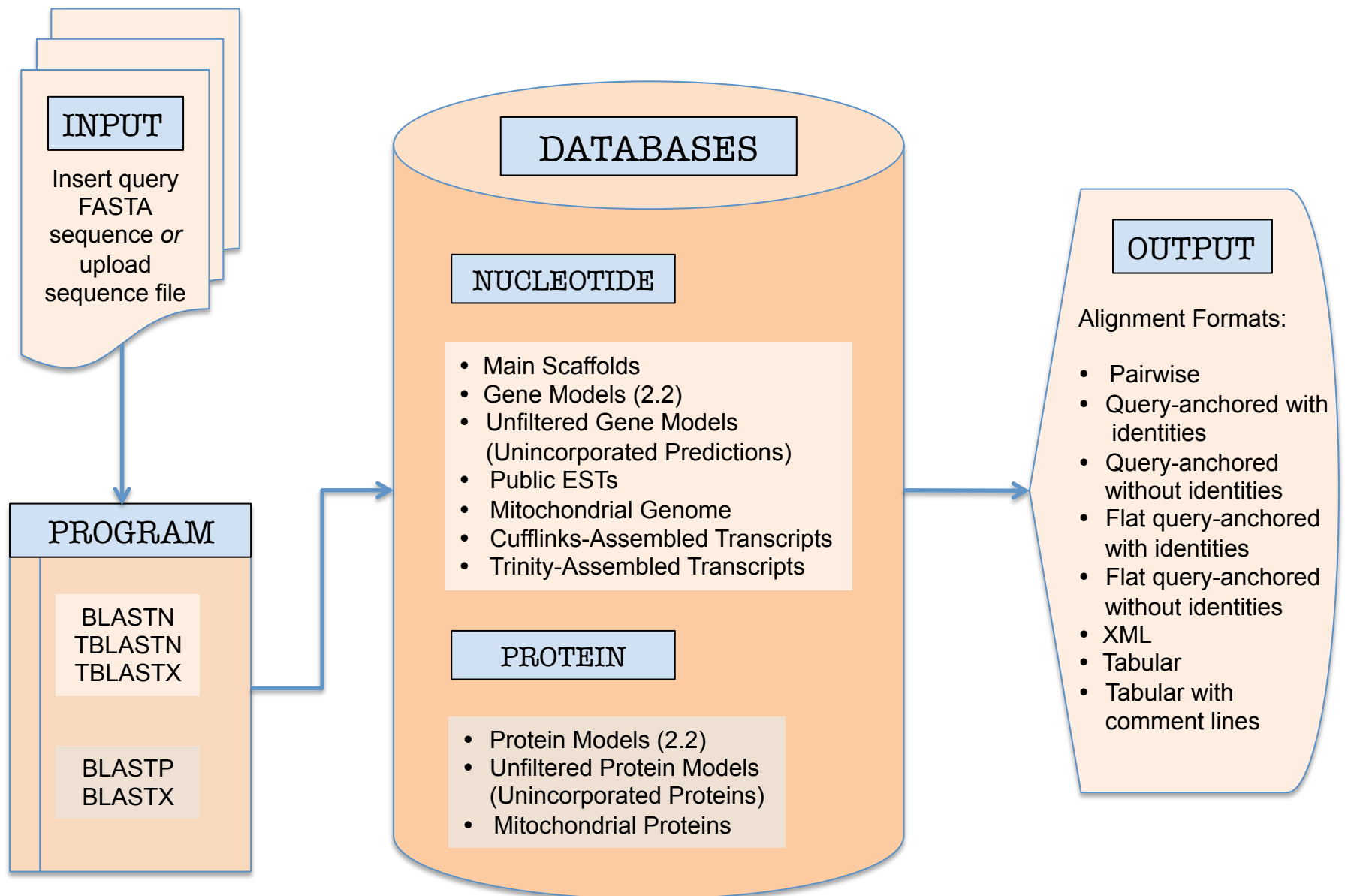

Supplement: Additional file 5: Figure S1 — The Mnemiopsis BLAST tool (implemented using ViroBLAST) schematic illustrates the available user-defined input and output formats, BLAST programs, and database options. BLAST databases are provided for both Mnemiopsis nucleotide (e.g., Mitochondrial genome) and protein [e.g., Protein Models (2.2)] data. [file 1471-2164-15-316-S5.pdf]

# KEGG

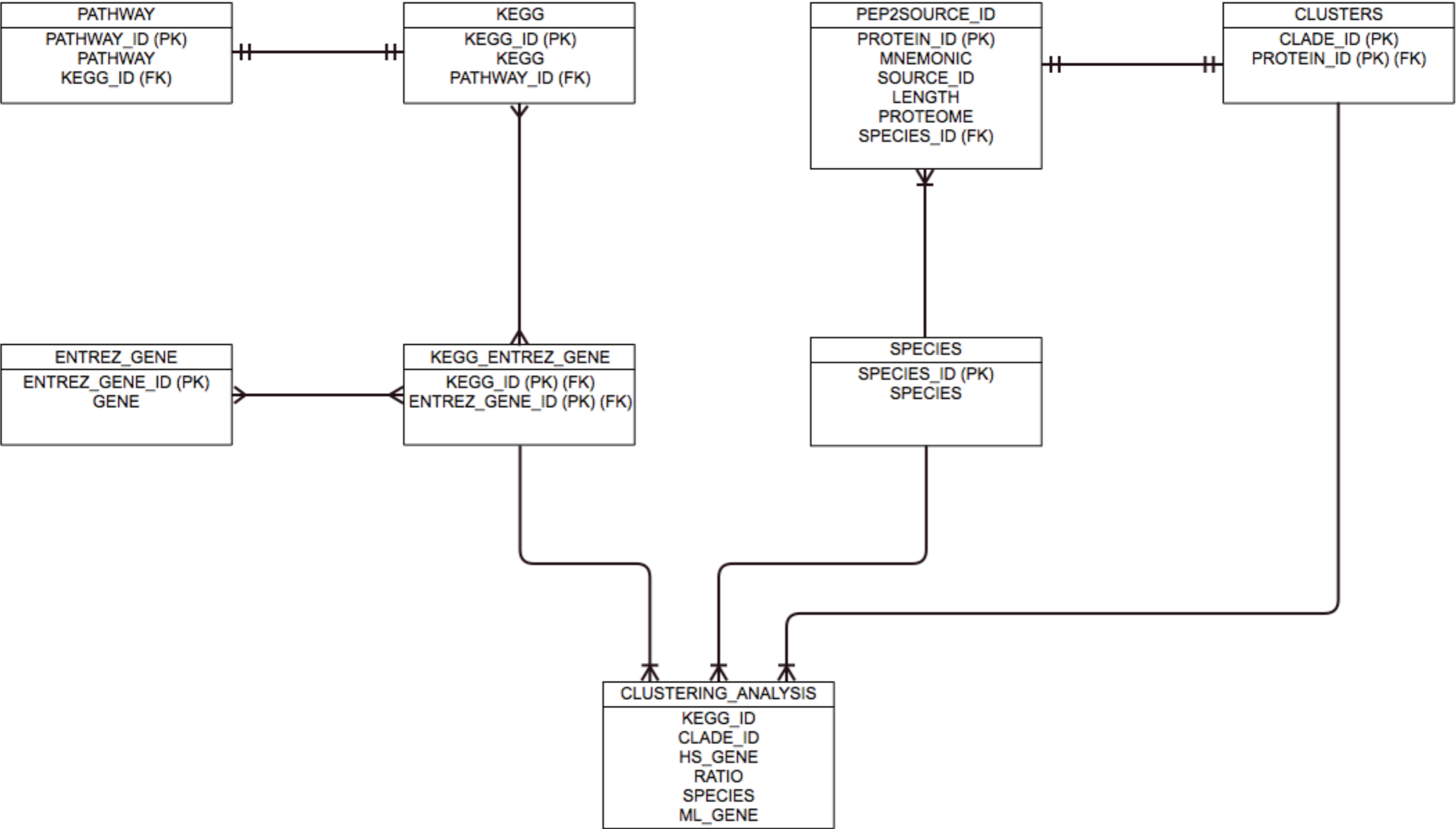

Supplement: Additional file 6: Figure S2 — The KEGG Pathways search function permits users to search KEGG pathways containing human genes, using a Mnemiopsis homolog as the query. The relationships underling the search function are depicted as a series of associated flat files. A one-to-one relationship exists between the KEGG and PATHWAY tables and the PEP2SOURCE_ID and CLUSTERS tables. All other relationships are one–to-many or many-to-many. The CLUSTERING_ANALYSIS table is the final output representation of a KEGG Pathways query consisting of the combination of KEGG_ENTREZ_GENE, SPECIES, and CLUSTERS. [file 1471-2164-15-316-S6.pdf]

# PFAM DOMAINS

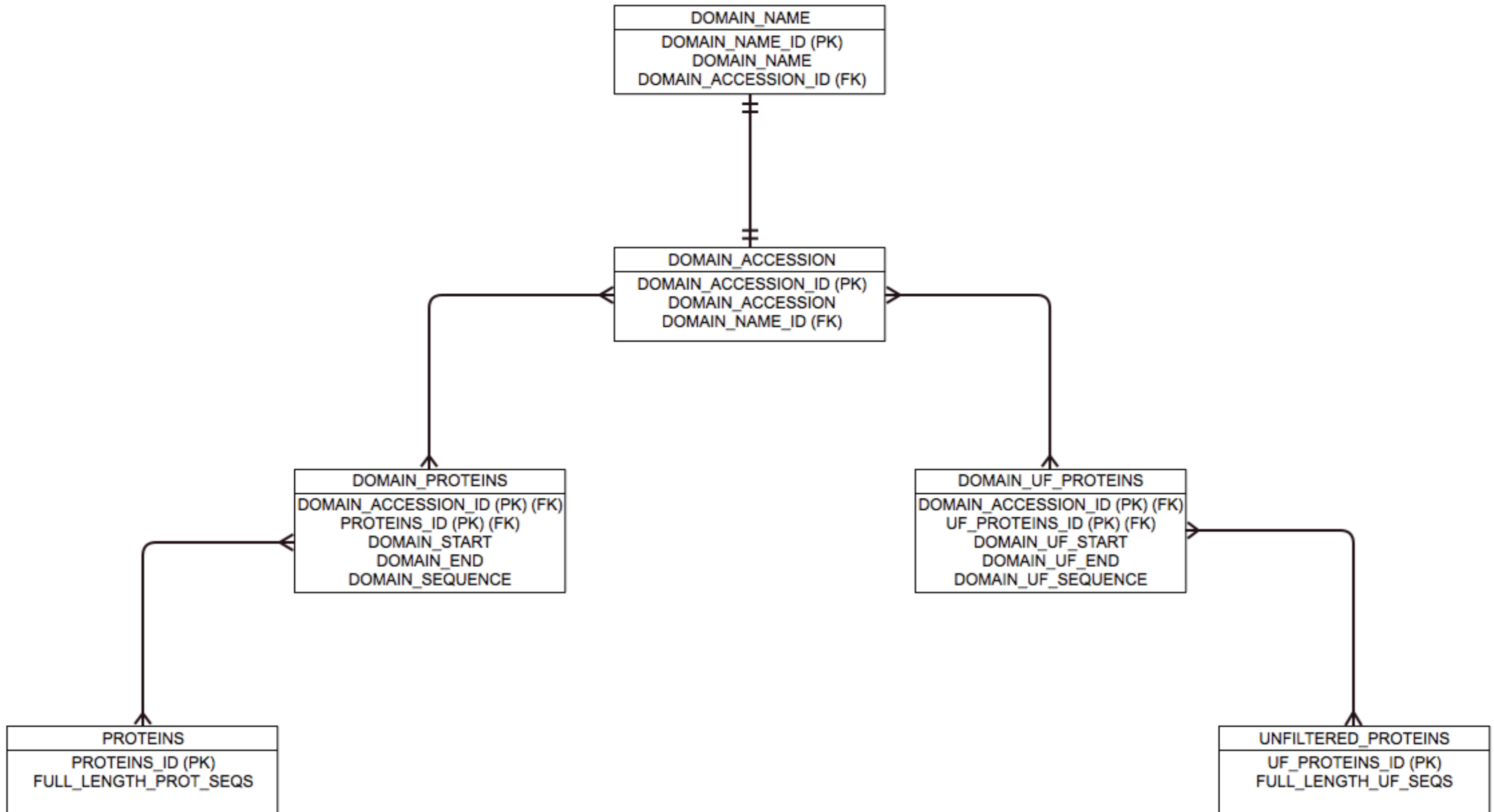

Supplement: Additional file 7: Figure S3 — The PFAM Domains search function parses a series of flat files illustrated here as a relational framework. The PFAM Domains schema is represented as six attributes, with connectors indicating the nature of each applicable relationship. DOMAIN_ACCESSION and DOMAIN_NAME have a one-to-one relationship. All other relationships between PFAM attributes are many-to-many. [file 1471-2164-15-316-S7.pdf]
